# Modelling of Cetylpyridinium Chloride Availability in Complex Mixtures for the Prediction of Anti-Microbial Activity Using Diffusion Ordered Spectroscopy, Saturation Transfer Difference and 1D NMR

**DOI:** 10.3390/ph17121570

**Published:** 2024-11-22

**Authors:** Cameron Robertson, Sayoni Batabyal, Darren Whitworth, Tomris Coban, Angharad Smith, Alessandra Montesanto, Robert Lucas, Adam Le Gresley

**Affiliations:** 1 Department of Chemical and Pharmaceutical Sciences, Faculty of HSSCE, Kingston University, Kingston-upon-Thames KT1 2EE, UK; tomriscoban@gmail.com (T.C.); a.legresley@kingston.ac.uk (A.L.G.); 2 Haleon, Weybridge KT13 0NY, UK; sayoni.x.batabyal@haleon.com (S.B.); darren.x.whitworth@haleon.com (D.W.); angharad.smith@rfi.ac.uk (A.S.); alessandra.x.montesanto@haleon.com (A.M.); robert.a.lucas@haleon.com (R.L.)

**Keywords:** NMR, formulation, anti-microbial, micelles, diffusion, cetylpyridinium chloride

## Abstract

**Background/Objectives:** A range of NMR techniques, including diffusion ordered spectroscopy (DOSY) were used to characterise complex micelles formed by the anti-microbial cationic surfactant cetylpyridium chloride and to quantify the degree of interaction between cetylpyridium chloride and hydroxyethyl cellulose in a variety of commercially relevant formulations as a model for the disk retention assay. **Methods:** This NMR-derived binding information was then compared with the results of formulation analysis by traditional disk retention assay (DRA) and anti-microbial activity assays to assess the suitability of these NMR techniques for the rapid identification of formulation components that could augment or retard antimicrobial activity DRA. **Results:** NMR showed a strong ability to predict anti-microbial activity for a diverse range of formulations containing cetylpyridinium chloride (CPC). **Conclusions:** This demonstrates the value of this NMR-based approach as a rapid, relatively non-destructive method for screening commercial experimental anti-microbial formulations for efficacy and further helps to understand the interplay of excipients and active ingredients.

## 1. Introduction

### Rationale

Quantifying and understanding the mechanism of antibacterial activity in non-alcohol mouthwashes has become a priority in formulation chassis design and IP creation for consumer health companies in recent years through greater understanding and consumer awareness of health concerns related to alcohol and paraben-based mouthwashes [1,2]. Accurate determination of anti-bacterial activity is required not only for appropriate advertising standards for the producer but also where the relative level of dental plaque reduction is determined through minimising non-native biofilm generation [3,4]. If this is not mediated, bacterial species that incorporate into the biofilm over time can lead to pathogenicity and oral health degeneration [5]. Standard mechanical oral care does aid in preventing generation of pathogenic bacteria, but proper management of the oral biofilm is also dependent on secondary considerations including diet, lifestyle and general health, where a diet high in sugar (athletes, physical workplaces, poorer countries, etc.) requires individuals to manage their oral health more carefully than those who have a more sedentary lifestyle and do not consume caffeinated, carbonated and sugary beverages or foods [6]. All these variables mean that different oral health products need to be available to manage unique oral health states [7]. The need for niche, personalised oral health products means that oral formulations are extensively and frequently modified to achieve the greatest results for the consumer. However, despite clinical trials and theoretical understanding of mouthwashes showing potential for new oral formulations, testing is required for different marketplaces for claim support. The main marketing statements for oral health products are either antibacterial based through specific action of an active ingredient within the formulation or through holistic action of the formulation on maintaining oral health [8]. The assays to evidence these claims are, however, not universal and are based upon different physicochemical properties of the active ingredients and their targets. Therefore, a general understanding of the specific action of active ingredients is predicated on its physicochemical properties in situ for different formulation types; for example, CPC acts in different bactericidal ways depending on its concentration [9].

The disk retention assay is a method used to capture charged CPC species, which can then be rinsed to quantify CPC [9]. This acts as a model of the surface of a tooth or biofilm; however, due to the different states CPC can exist in (free, micelle, vesicle, etc.) with different excipients, the DRA assay can potentially erroneously indicate lower levels of CPC in formulations that have bactericidal potential. Therefore, products that perform well in bactericidal specific assays will not see correlation to DRA results [10,11,12]. The abundance of freely available CPC_f_ is important to consider, as its anti-bacterial activity is dependent upon the cationic nitrogen being able to interact with the bacterial cell membrane and cause disruption leading to cell death of the bacteria through various mechanisms [9,13,14,15]. However, CPC is a surfactant, and at the target concentrations for high antibacterial activity in a simple aqueous solution, it will pass the critical micelle concentration (CMC) and form micelles (CPC_m_) or other secondary structures. When alcohol is present, this does not occur, as intermolecular bonds between CPC molecules are disrupted; however, in these cases, most antibacterial activity is conveyed by the alcohol rather than the CPC [15,16]. Consequently, manufacturers of these formulations aim to increase the CMC of CPC to give a greater pool of free CPC (CPC_f_) compared to micellised CPC (CPC_m_) or other secondary forms of micelle, e.g., vesicular, polymersomic, etc. (CPC_2_^o^). The DRA test is reliant upon the ion-hydrogen bonding of cationic CPC_f_ to the anionic surface of the cellulose disk; therefore, any other forms of CPC (CPC_m_/CPC_2_^o^) will potentially be filtered through with the bulk of formulation [17,18,19]. The CPC can also form polymeric micelle systems within formulations if interactions exist between the CPC and emulsifiers used within the system, leading to an increase of CPC in the form of CPC_2_^o^ even when the CMC of CPC is not reached, either through a lower CPC or through addition of excipients that are known to increase the CMC [20,21]. The value of CMC has been shown to be affected by introduction of ionic species, excipients, pH and the temperature used to prepare formulations. Whilst antimicrobial activity correlates to the amount of CPC that is introduced, the DRA test shows sensitivity to the macromolecular forms of CPC [22]. If the CPC has its dipole moment reduced, it will not be retained to the same extent on the cellulose disk as monomeric CPC that retains its large dipole [23,24,25]. Therefore, NMR can be used to not only physiochemically characterise CPC but also identify the excipient compounds that CPC is interacting with, its micellation state and, to a lesser degree, its electrostatic potential through negative chemical shift changes for pyridine peaks as the quaternary ammonium group interacts with hydroxy groups present in block copolymers, glycerol and anionic excipients [25].

This is a problem when designing a new formulation, as the only way to discover the problems in the formulation is through step-wise removal/addition of excipients in a very time-consuming and materially expensive manner. Understanding the distribution of population forms of CPC can be as important as quantifying the active CPC within the formulation [20]. Understanding the physicochemical nature of CPC in formulation and quantifying CPC_f_ can give a fuller picture to the new product development scientist, where excipient effect, concentration and relative stability can be explored, and more informed formulation decisions can be made based upon physicochemical understanding rather than end-point antibacterial activity data. Alongside this understanding, it will allow for greater pipeline management and allow for progression to next steps in product development with confidence that specific excipient additions and removals will not have a significantly negative effect [19,20].

The goals of this research are to evaluate the application of NMR techniques for the characterisation of CPC and its interactions with excipients in both full commercially equivalent formulations and experimental simple combination formulations. Of particular interest were the effects of block copolymers/emulsifiers (Supplementary Information S11), preservatives/flavourings (Supplementary Information S2) and ionic strength on CPC availability and physicochemical state.

To achieve these aims, characterisation of CPC was undertaken with a suite of 1D and 2D NMR techniques, including diffusion ordered spectroscopy (DOSY) and saturation transfer difference (STD) NMR in full and experimental CPC formulations. DOSY allows for the separation of species based upon calculated diffusion coefficients in solution; this can then be used to interpret the physicochemical state of molecules of interest and infer effects of excipients on actives. STD directly demonstrates interaction through the transfer of frequency saturation from a larger target molecule to smaller ligands of that target. This transfer can then be quantified to give relative measures of interaction strength and localisation. The analysis of the data acquired allowed for the assessment of the impact of excipients on total solubilised concentration of CPC and the amounts of CPC_f_ versus other states through the degree of micellisation being averaged directly by DOSY NMR. The use of DOSY and qNMR data to establish the ratio of CPC_f_ to CPC was further tested in a novel assay where binding to soluble hydroxyethyl cellulose (as a water-soluble cellulose mimic for the DRA) was evaluated to determine correlation to anti-microbial activity and DRA assay. Determining levels of signal attenuation and line-broadening in ^1^H NMR signals for CPC before and after introduction of hydroxyethyl cellulose gave information on the relaxation properties of CPC, which links to a change in relative motion/tumbling/mobility in solution, impacting the kinetics of any CPC interaction.

The research aims are indicated below.

(A) Full formulations (Section 3.1)
  1. Excipient interactions, e.g., block copolymers.
  2. CPC diffusion NMR correlation with disk retention assay and antimicrobial activity.
  3. Solubilised hydroxyethyl cellulose as a potential DRA mimic.
(B) Experimental formulations (Section 3.2)
  1. Using PCA to evaluate different excipients to better understand the mixed micellisation and availability of CPC.
  2. Establishing impact on observed diffusion of CPC of a broad range of experimental formulations.
  3. Using CPC NMR diffusion data to optimise availability and antimicrobial activity of CPC.

## 2. Results and Discussion

### 2.1. Full Formulation

#### 2.1.1. Representative Excipient Interactions—Block Copolymers, Parabens and Phosphate

Diffusion coefficient values for CPC aromatic NMR signals in different formulations give a relative comparison of hydrodynamic radius for CPC states, from free monomeric CPC_f_ and micellised CPC_m_ to incorporation of CPC into larger block copolymer-derived structures (e.g., CPC_2_^o^) [26]. An expanded uncertainty of ±1.2% was determined for diffusion coefficient values calculated for “simple” CPC and additional excipient formulations with an expanded uncertainty of ±1.6% determined for full and commercial formulations (expanded uncertainty k = 2) with the settings used throughout study.

One initial aim was to observe the impact on CPC NMR signals of the preservatives methyl 4-hydroxybenzoate (methyl parabens) and propyl 4-hydroxybenzoate (propyl parabens) (see structures in Supporting Information S2). Using a simple system of CPC, block copolymer and either paraben, DOSY NMR indicated that both preservatives had a pronounced effect on the average CPC diffusion and hence the size of the micelles formed. Whilst the decrease in diffusion seems small, as this is a logarithmic scale, this actually translates to a four-fold increase in the CPC micelle volume. The chemical shifts of the signals relate to the pyridinium ring protons indicated in the CPC structure in Supporting Information S1, where H_1_ = 8.9 ppm, H_3_ = 8.6 ppm and H_2_ = 8.1 ppm.

**Figure 1:**
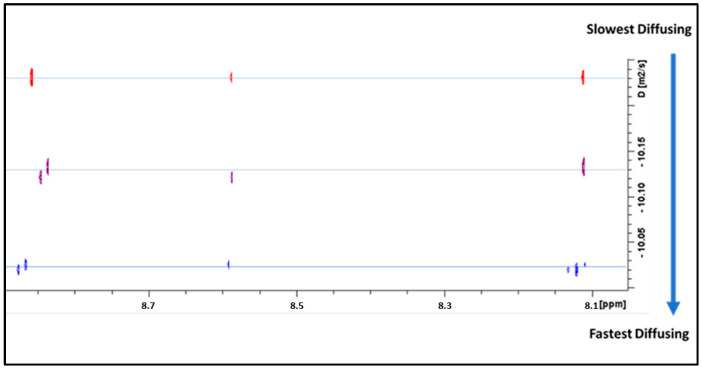
DOSY NMR spectrum (chemical shift versus Log_10_ of diffusion coefficient (m^2^s^−1^) for 0.07% *w*/*w* CPC in the presence of (**top**) P407 block copolymer (3 mg/mL), (**middle**) methyl parabens (0.5 mg/mL) and P407 block copolymer (1 mg/mL), (**bottom**) P407 block copolymer (1 mg/mL)). Diffusion was referenced to TPS as the internal standard.

Based on the observation of direct involvement of the above preservatives in micelle formation and changes to hydrodynamic radii observed by DOSY NMR (Figure 1), STD NMR was employed to attempt to discern the degree and orientation of binding of both paraben species to CPC. Via irradiation of the aliphatic chain of the CPC at a PPM (~1 ppm) avoiding the aliphatic elements of parabens, it is apparent that the interaction of the parabens with the pyridinium ion of the CPC shows a preference for a specific orientation on the surface of the CPC micelle [27].

**Figure 2:**
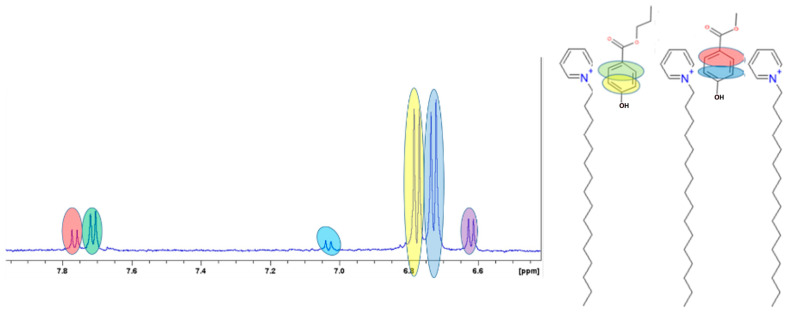
(**Left**) STD NMR difference spectrum (*x*-axis chemical shift (ppm) spectrum with downfield (8 ppm) signals for both parabens significantly less attenuated compared with the upfield signals (7.5 ppm) for the same molecules representing greater saturation transfer). (**Right**) Representation of the relative orientation of the parabens with respect to the CPC monomers forming a micelle as hypothesised from STD data. The different colours correlate to the proton environments, which are assigned. Blue and purple residual signals are from anethole.

This is evidenced in Figure 2 (left) where the signal attenuation of the aromatic signals proximal to the ester substituent is more pronounced than that for the signals proximal to the OH group. The qualitative significance is that the signals with the lower difference after saturation are further away from the irradiated molecule. The results therefore suggest an arrangement as represented by Figure 2 (right), where the proximity of each paraben can also be compared. The reason for the screening of saturation transfer at the aromatic peaks is due to not only their visibility in model mixtures but their potential to be resolved in complex mixtures as well.

**Figure 3:**
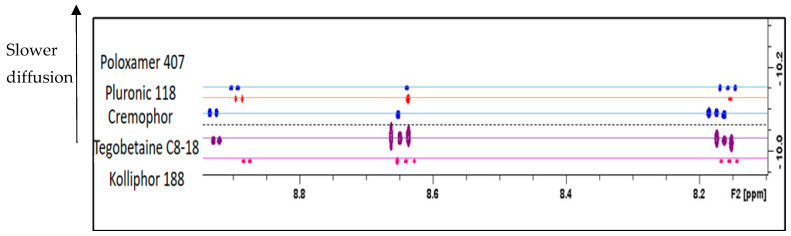
DOSY NMR (chemical shift versus log of diffusion coefficient (m^2^s^−1^) of CPC aromatic signals for different block copolymers). CPC/MP/PP (2 mM) and block copolymer (5 mg/mL). This shows the combined effects of parabens and block copolymer types on average CPC micelle size.

The effects of block copolymers on CPC micelle size were also investigated using DOSY NMR, and it was apparent that there is a substantial impact of the nature of the block copolymer on the micelle size (Figure 3). It is noteworthy that this is not a general viscosity effect, as this was corrected for by the water soluble internal standard TSP.

**Figure 4:**
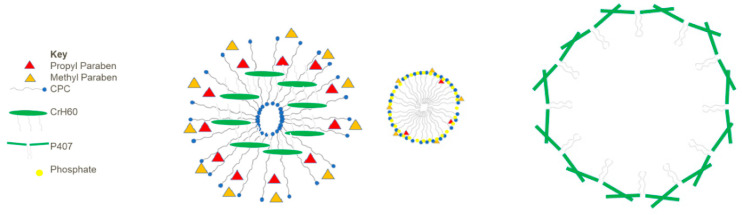
Simplistic cartoon representations of the interactions between CPC and block copolymer. (**left**) Formation of mixed Cremophor and parabens/CPC liposome and (**middle**) the formation of discrete CPC micelles in the presence of phosphate/lower parabens concentrations and (**right**) isolated P407 block copolymer as determined by NMR techniques described previously.

What became apparent from the DOSY/STD NMR data was the synergistic effect of the parabens and the block copolymer in terms of the ultimate state/size of CPC micelles. Based on changes in diffusion of the CPC in the presence and absence of both parabens and the block copolymer, it was possible to deduce that for certain block copolymers (e.g., Cremaphor), a large mixed micelle of CPC was forming, with little CPC_f_. For other co-blockc polymers, e.g., P407, with a lower concentration of parabens, the interaction with CPC was minimal, facilitating discrete CPC micelle formation and increasing the effective CPC_f_ (Figure 4) [28].

This interplay between the excipients was confirmed by the introduction of phosphate. It is well established that phosphate adsorption onto cationic surfactant preparations reduces the curvature of micelles formed, therefore reducing the respective size of CPC_m_ formed. This also reduces the forces holding CPC into the structures and may increase CMC for CPC_m_. The addition of 0.025 M PBS in the presence of 0.07% CPC gave a substantial increase in diffusion rate for CPC, with a subsequent decrease from this optimum value as the PBS concentration was increased (Supporting Information S3). It is unsurprising that introduction of PBS after first adding parabens to CPC results in the parabens being lost by the CPC, as the sparing solubility of both leads to precipitation. It is therefore important to consider lower phosphate concentrations to maintain the equilibria of solubility [29].

#### 2.1.2. Hydroxyethyl Cellulose (HEC) as a DRA Proxy for Full Formulations

**Figure 5:**
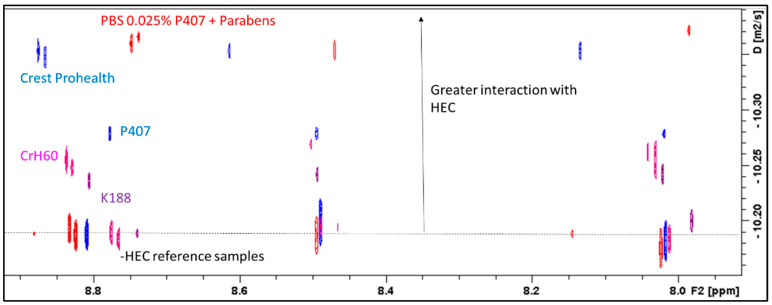
2D ^1^H DOSY NMR, illustrating the impact that the block copolymer has on the interaction of the CPC (aromatic signals only) with HEC. The bottom line shows the CPC diffusion for reference samples prior to HEC addition.

As discussed in the introduction, the DRA is used to determine the availability of CPC in a given formulation based on an affinity to a cellulose disk. It was hypothesised that based on the sensitivity of CPC diffusion to formulation composition, it would be possible to substitute this assay with a partially water-soluble cellulose mimic [26,27]. Hydroxyethyl cellulose (HEC) was selected as the proxy for the DRA, and DOSY NMR data for CPC in the presence and absence of HEC w evaluated (Figure 5).

It is particularly noteworthy that the shift in CPC diffusion is less pronounced with the K188 and Cremophor block copolymers in the formulation. This underlines the conclusions from the previous sections that mixed micelles of CPC and block copolymer reduce the amount of CPC_f_ [30,31].

#### 2.1.3. Evaluation of DOSY NMR Signals for CPC Compared with DRA/Antimicrobial Data

**Figure 6:**
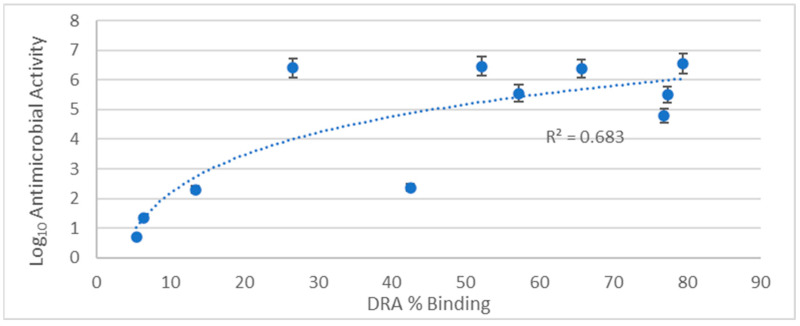
Log_10_ antimicrobial activity of *E. coli* vs. DRA result as % binding.

With the full formulation samples detailed in Section 3.2, the correlation between the DRA and antimicrobial activity is shown in Figure 6. The correlation of these two data sets for the full formulations did not appear as linear as might have been expected, suggesting that while it is a standard method, the DRA results showed considerable variability and a poor fit overall to the Log_10_ antimicrobial activity. This, based on our discovery of the many and various interactions of the excipients with CPC, is unsurprising and suggests that the DRA does not perhaps represent the concentration of CPC_f_, which should have a direct correlation with Log_10_ but rather some ratio of [xCPC_f_ + yCPC_m_].

**Figure 7:**
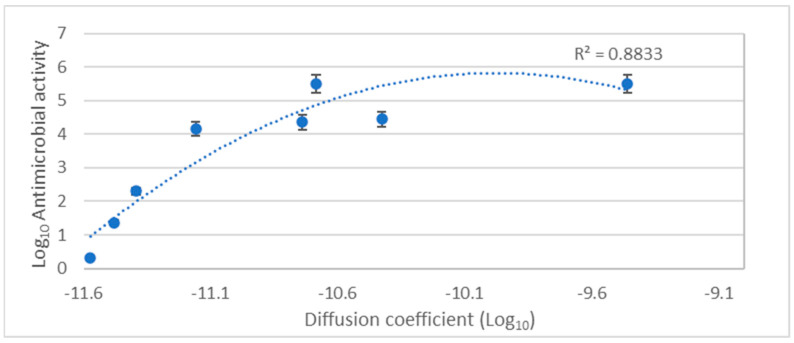

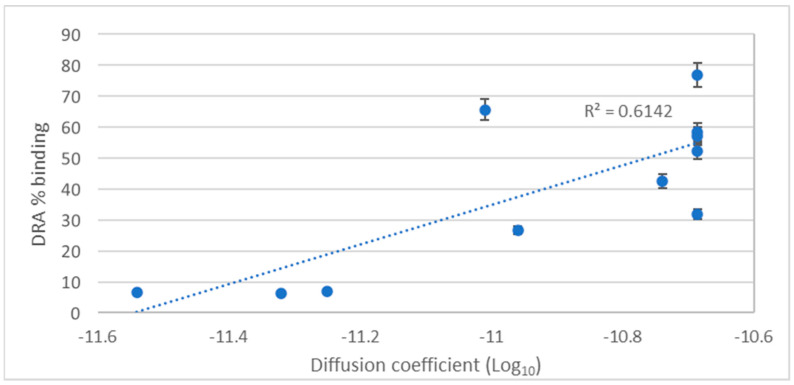
(**Top**) Diffusion coefficient from DOSY NMR for CPC aromatic signals vs. Log_10_ antimicrobial activity against *E. coli*—polynomial trendline. (Bottom) DRA % binding score vs. diffusion coefficient from DOSY NMR for CPC. These full formulations have been anonymised in the graphs.

Comparison of DOSY NMR data with antimicrobial activity and DRA values (Figure 7) demonstrates a correlation between diffusion coefficient and both antimicrobial activity and DRA. However, it is noteworthy that the r^2^ values, whilst better than those for Figure 6, are still quite poor, especially for the diffusion coefficient/DRA correlation. More compelling is the apparent correlation between diffusion coefficient and antimicrobial activity, where the faster diffusion constant reflects the average diffusion for CPC based on [xCPC_f_ + yCPC_m_]. The average diffusion parameter increasing reflects an increase in the x term and a decrease in the y, indicating “freer” CPC to engage in antimicrobial interactions. This suggests that DOSY NMR represents a potential method for screening complex, non-alcohol-based mouthwashes in a relatively non-destructive manner for antibacterial activity.

#### 2.1.4. Considering Other CPC NMR Signal Characteristics in the Presence and Absence of HEC

**Figure 8:**
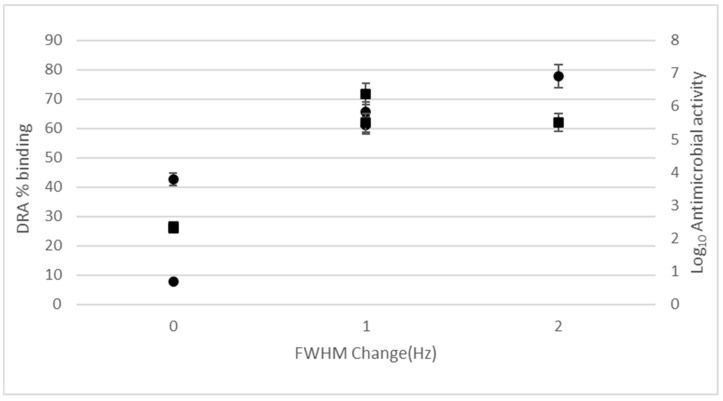

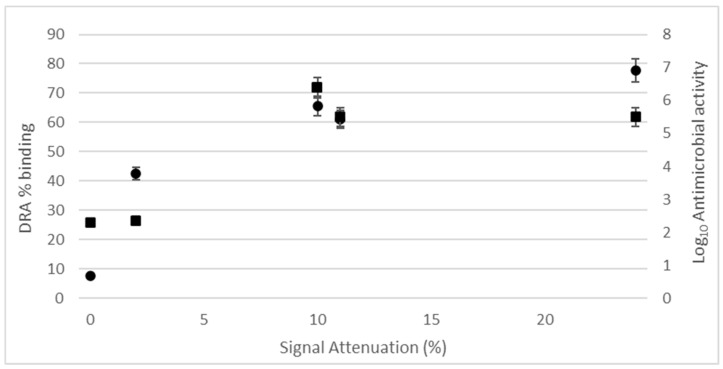
(**Top**) DRA (●) and antimicrobial activity (■) vs. change in full width at half maximum (FWHM) after addition of HEC (10 mg/mL). (**Bottom**) DRA (●) and antimicrobial activity (■) vs. signal attenuation after addition of HEC (10 mg/mL).

As discussed in the introduction, the DRA model is used to predict antimicrobial activity based on the binding of the cationic pyridinium “head” of CPC with a cellulose disk at the liquid/solid interface. Owing to the dimensionality of NMR, the intensity and line shape of the ^1^H NMR signals for CPC were also considered, not just to indicate the relative amounts of CPC in solution (as opposed to precipitated or held as a suspension by excipients) but also to use qualitative consideration of line-broadening (indicative of T_2_^*^) as a measure of in situ isotropy of the molecule itself. The HEC-induced signal attenuation and change to full width at half maximum (FWHM) in an aqueous environment for the three aromatic CPC resonances were compared for the full formulations (Figure 8).

Reference to Figure 8 shows correlation of signal attenuation and NMR signal broadening after HEC addition for both DRA and anti-microbial activity and reflects not just the concentration of CPC but the degree of micellisation. In both cases, there is not a reliable linear relationship, with a plateau after either a 1 Hz line broadening or 10% signal attenuation [32].

### 2.2. Experimental Formulations

#### 2.2.1. Antimicrobial Activity vs. Available CPC Based on qNMR and Diffusion Parameter

Building on this work, the practical application of NMR to predict antimicrobial activity of CPC-based formulations required expansion, and experimental formulations were prepared as indicated in Section 3.3. Based on the previous analysis of interactions of excipients and outlier values, it is clearly also important to consider not just the effective micelle size of CPC but also the actual [CPC]_f_. The base formulation of any mouthwash will doubtless have an impact on the apparent diffusion and available [CPC}_f_. To convert the abstract diffusion parameter into a more recognisable format, the binding of CPC to HEC was determined using the method of Fielding et al., which relates observed diffusion for a 1:1 binding isotherm, to be that shown in Equation (1) [33].

D_obs_ = X_G_D_G_ + X_HG_D_HG_ (1)

which, when the assumption is made that the diffusion of HEC remains unchanged when bound to CPC, becomes Equation (2).

K_a_ = X_HG_/((1 − X_HG_)[H]_o_ − X_HG_[G]_o_)) (2)

**Figure 9:**
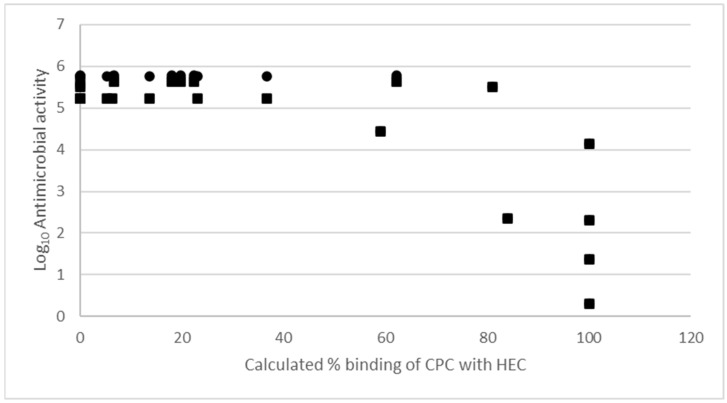
Experimental formulations calculated binding (Equation (3)) of CPC with HEC vs. antimicrobial activity. (■) S. mutans (●) *E. coli*. Formulations detailed in Table S1a.

This enables the direct approximation of K_a_ and hence the % of CPC bound to HEC. Using this, it was possible to show a relationship between HEC-CPC binding and antimicrobial activity, which indicates that beyond a certain degree of HEC binding, antimicrobial activity decreases, but if the NMR assay shows binding below 50%, a high level of antimicrobial activity is still possible. This is unsurprising, as the correlation between DRA and antimicrobial activity is quite poor, as shown in Figure 6. Our data show that that highly HEC-bound CPC generally showed poor availability and hence poor antimicrobial activity. At first sight, the relationship in Figure 9 would appear to be at odds with the standard DRA methods. However, since our results so far imply that smaller CPC units are better for antimicrobial activity, this highlights the differences between the two methods. The HEC method considers isothermal binding in a largely aqueous environment, accounting for the different average CPC micelle sizes and the DRA model, which looks at the liquid/solid interface and does not allow discrimination between CPC micelle sizes. This highlights the limitation of directly comparing the HEC and DRA methods.

Whilst the addition of HEC to mimic DRA in situ was a rational approach, the actual correlation between DRA and antimicrobial activity is far from ideal, so the focus was turned to correlating NMR signal intensity/diffusion for CPC directly to antimicrobial activity across the range of experimental samples as indicated in Section 3.3.

**Figure 10:**
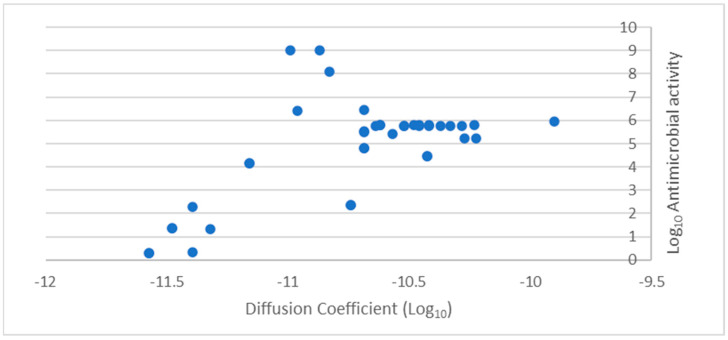
Clustering of Log_10_ antimicrobial activity (*E. coli*) vs. diffusion parameter for CPC. This combines Table S1a–c.

Hence, the CPC diffusion values in the absence of HEC were considered alone, in terms of antimicrobial activity (Figure 10), which showed a solid correlation with a few outliers where the concentration of [CPC]_f_ was higher, further endorsing the premise that a combination of factors can increase or decrease the CPC activity.

**Figure 11:**
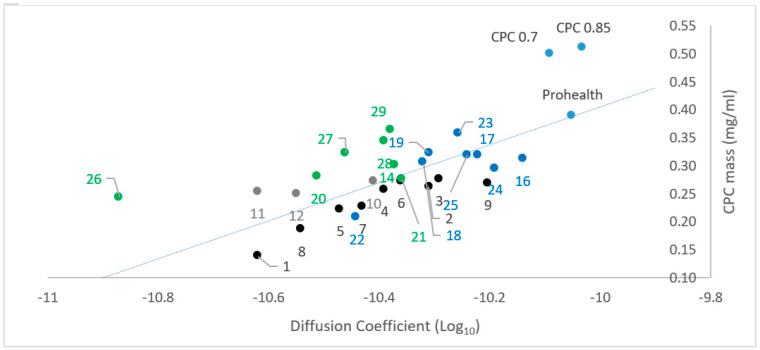
qNMR determined concentration of CPC vs. diffusion coefficient for CPC. Full formulation from Section 3.2 used as a point of reference (Table S1b). The different colour groupings indicate different batches as per Table S1a–d.

To reconcile the average observed diffusion coefficient of CPC_f_ with [CPC]_f_, these values were compared for experimental samples, as indicated in Section 3.3 (Figure 11). This shows a correlation between the two variables and comparison with a solution containing only CPC (CPC 0.7 and CPC 0.85) to identify trends for the formulation. Interestingly, it was possible to prepare a series of formulations for a given basic chassis and show a correlation between the [CPC]_f_ and the diffusion coefficient for CPC. This essentially allows for experimental formulations to be assessed by NMR to see how close the values are to the idealized CPC data points and full formulation labelled below.

**Figure 12:**
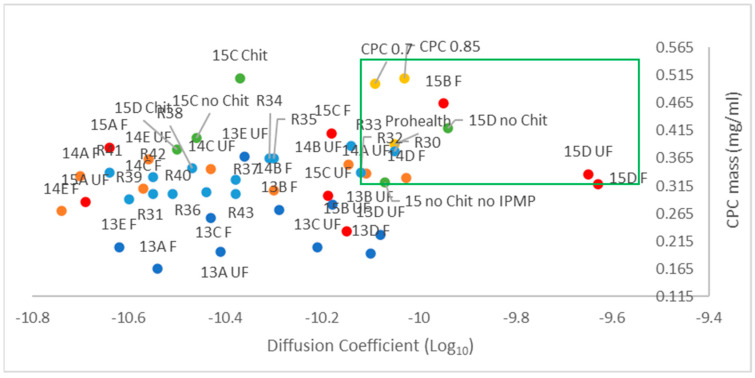
qNMR determined concentration of CPC vs. diffusion coefficient for CPC for wider range of experimental formulations, with green box to indicate the “ideal” zone for CPC. This combines data from Table S1a–d. The different colour groupings indicate different batches as per Table S1a–d.

Using the principle that high [CPC]_f_ with a low [CPC]_m_ is desirable for a mouthwash; it was possible to use this data to identify a region for a given experimental sample that could be predicted to have acceptable (i.e., >5) antimicrobial activity (experimental formulations used for this are indicated in Section 3.3). This region was shown to correlate well with subsequent antimicrobial activity measurements and demonstrates the application of NMR in predicted the efficacy of CPC-containing formulations (Figure 12).

#### 2.2.2. Hydroxyethyl Cellulose (HEC) as a DRA Proxy for Experimental Formulations

With HEC acting as a cellulose mimic, the impact of HEC on diffusion of CPC should indicate the degree of binding likely to occur between the CPC in both micellised and free forms and cellulose from the DRA. This should consider the different block co-polymer chassis components and the mixed micellization that becomes possible with CPC, including flavouring.

**Figure 13:**
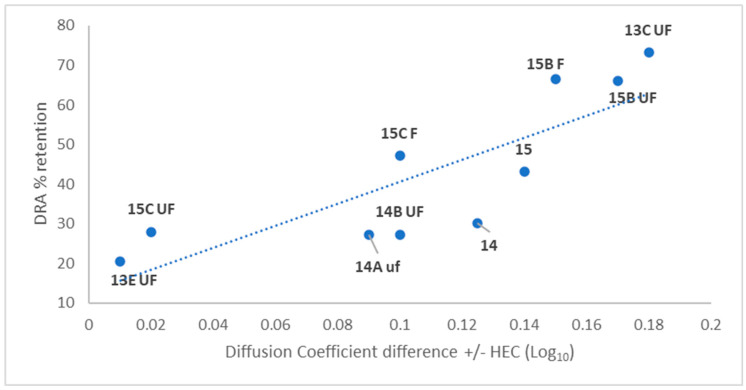
DRA vs. diffusion coefficient change (Log_10_) for HEC-adsorbed CPC signals for wider range of experimental formulations. Taken from Table S1c. UF indicates an unflavoured version of the same numbered formulation.

Figure 13 indicates the correlation between the change in diffusion parameters upon addition of HEC (10 mg/mL) (see Section 3.4 for method details) and shows a positive correlation with DRA values. The fact that these are simpler models may be the reason for this correlation and highlights the impact that formulation complexity has on this relationship (see Figure 7). It also indicates that the flavouring component does not distort the relationship between DRA and CPC diffusion differences when HEC is added.

#### 2.2.3. PCA Models for Experimental Formulations

The PCA of the variables shown in Table 1 was carried out as stipulated in Section 3.8 with normalisation performed first. The loadings plot highlights the diverse range of experimental formulations prepared (all 114 experimental formulations (Table S1a–d), but it is noteworthy that unsurprisingly the initial CPC concentration and [CPC]_f_ are correlated positively with antimicrobial activity (MK), and there is an inverse correlation between antimicrobial activity and absolute diffusion of CPC resulting from the diffusion constant being based on Log_10_ values that are negative. Hence, the larger, more negative values correlate to larger CPC micelles as in Figure 10. As indicated in Figure 7, there is a partial correlation between MK and DRA, and the HEC attenuated diffusion coefficient also sits in the same quadrant as the DRA parameter, but it is clear from the scores plot (Supporting Information S4) that the interplay of excipients with CPC can impact the validity of the DRA value as a proxy for antimicrobial activity.

**Figure 14:**
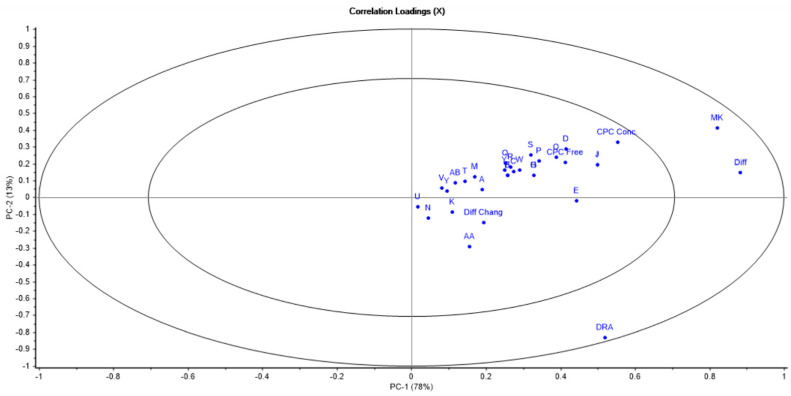
PCA loading plot for 114 experimental formulations. See Table 1 (Section 3.8) for legend.

Whilst the analysis is not exhaustive, there exists the possibility of predicting the antimicrobial activity of a given CPC formulation by considering the clustering of diverse experimental formulation groups (Figure 14) and of indicating experimental incompatibilities that could potentially streamline the formulation process for CPC-containing mixtures, achieving the desired activity within a shorter timeframe, with fewer experimental formulations being intrinsically outside of specification.

## 3. Materials and Methods

### 3.1. General

Volumetric measurements were undertaken using VWR (Soulbury, UK) single-channel mechanical, variable volume, ergonomic high performance (EHP) pipettes. A Mettler Toledo MT5 scale (Metler Toledo UK, Leicester, UK) was used for solid state mass measurements.

### 3.2. Full Formulations Used

Formulations supplied by GSK/Haleon September 2020:

As these are proprietary, full composition details are not available beyond the below.

**Therastorm**

CPC 0.075% calcium EDTA, essential oils, glycerin, PEG-40, poloxamer 407, propylene glycol, sodium benzoate, zinc, xylitol, gluconate.

**ProHealth clinical**

CPC 0.1%

Glycerol, hydrogen peroxide, flavor, sucralose, poloxamer 407.

**Zero**

CPC 0.075%, glycerin, propylene glycol, sorbitol, poloxamer 407, potassium sorbate, citric acid, rebaudioside A.

**ViJon HEB/Paradontax**

CPC 0.07%, glycerol, flavour, poloxamer 188, sodium saccharin, propylene glycol, sodium benzoate, sucralose, benzoic acid, blue 1.

### 3.3. Experimental Formulations Prepared

To cover the largest number of variables, 114 experimental formulations were prepared. A complete list of all formulations and their composition is available in Supporting Information Table S1a–d. Owing to the size of the spreadsheet, these data are in Supporting Information as a Microsoft Excel file. They are separated according to the section they are discussed in:

S1–S15 (Table S1a) discussed in Section 2.1 represent the full formulation but synthesised with different excipients to better understand the mixed micellization and availability of CPC.

F1–F41 (Table S1b) samples discussed in Section 2.2 (relating to Figure 11, Figure 12 and Figure 14) represent a broad range of experimental formulations.

F13A to F16J (Table S1c) samples discussed in Section 2.2 (relating to Figure 12, Figure 13 and Figure 14) represent a narrower range of formulations with only subtle variations. (A–J). These samples are also either flavoured (F) or unflavoured (no suffix).

LRH1 to LRH19 (Table S1d) samples discussed in Section 2.2 (relating to Figure 14) represent a range of formulations with differing zinc salts.

It is important to recognise that some of these commercial experimental samples could not all be analysed in the same manner, as this was an industrial-academic partnership, with certain data that is proprietary and cannot be published. The tables in Supporting Information show the full compositional details of all formulation and experimental NMR/DRA and antimicrobial activity data that could be obtained from the various batches of material supplied.

### 3.4. Sample Preparation

Mouthwash solutions were agitated for 3 min by a vortex mixer before 9 mL was removed by an Eppendorf pipette (VWR, Soulbury, UK) and put in a sample vial to which was added 1 mL of D_2_O with internal standard TSP (99.9 atom % D, contains 0.03 wt. % 3-(trimethylsilyl)propionic-2,2,3,3-d4 acid, sodium salt, Merck Life Science, Gillingham, UK) to enable locking of the NMR field and referencing of spectra.

An amount of 650 µL of this solution, containing the internal standard at 100 μM, was added to an NMR tube (as described in Section 2) to enable quantitation of formulation components and diffusion parameters.

The preparation of samples with hydroxyethyl cellulose added is discussed in Section 3.7.4.

Samples were then subjected to NMR analysis as per the below (Section 3.7).

### 3.5. Disk Retention Assay

The in vitro measuring of CPC is achieved using a cellulose filter disc that through binding of cationic CPC molecules to the anionic surface can determine abundance of free CPC. An important limitation of the DRA is that the response saturates at around 0.3% (CPC) with a linear response. It has been used to show the limiting effect of surfactants in oral health formulations [24]. The DRA method was performed using the literature method by pipetting mouthwash samples containing CPC on a stack of two pre-wetted cellulose disks, allowing them to equilibrate for 1 min, centrifuging the stack of disks, and measuring the CPC level in the supernatant by HPLC with UV detection at 265 nm.

### 3.6. M10 Time KillTime Kill (E. coli)

**Scheme 1:**
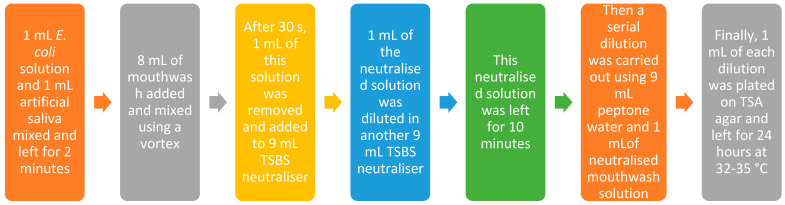
Method for M10 kill time test for antimicrobial activity for *E. coli*.

Solutions of 200 mL 0.07% *w*/*w* CPC and one or two other excipients were made up and tested with the M10 (M10 guidelines) time kill test using *E. coli*, with a 30 s contact time in artificial saliva with urea. The *E. coli* suspension was made up in peptone water to ~0.30 absorbance for M10 testing as per the protocol (Scheme 1).

Amounts of 1 mL *E. coli* solution and 1 mL artificial saliva were added together and left for 2 min before adding 8 mL of mouthwash solution, mixing and removing 1 mL after 30 s. This was added to 9 mL TSBS (tryptic soy bath solution) neutraliser and mixed, and then 1 mL of this was added to the next 9 mL of TSBS and mixed. After ten minutes, the serial dilution was carried out with 1 mL from the second neutralised solution being added to the peptone diluent, mixed and repeated for 3 dilutions. Then 1 mL of each dilution (−5, −4, −3, −2) was plated up with TSA agar on two plates for each dilution. The agar was mixed gently and left to set before placing in an incubator for 24 h at 32–35 °C. After 24 h, the colonies on the plates were counted and results calculated. Log_10_ antimicrobial activity is Log_10_ of the number of bacteria killed after the above process.

### 3.7. Nuclear Magnetic Resonance (NMR)

A Bruker Avance NEO 600 MHz FT-NMR Spectrometer (Bruker UK Ltd., Coventry, UK) at 300 K was used for all NMR acquisitions. NMR tubes used were 5 mm, Ultra-Thin Wall Precision NMR Sample Tubes 7″ L, 600 MHz, (545-PPT-7), from GPE-Scientific (Leighton Buzzard, Bedfordshire, UK). A variable temperature unit kept the temperature at 298 k for all samples. Unless otherwise stated, the number of scans (NS) for 1H 1D, DOSY 2D and STD experiments were 1024, 64 and 64, respectively, with relaxation delays determined by T1 inversion recovery experiments.

TopSpin 4.3.0 (Bruker UK Ltd., Coventry, UK) was used for acquisition preparation and processing of raw data. Automatic Fourier transformation, phasing and baseline correction were applied followed by manual phase correction and manual integration of peaks with bias and slope manually corrected.

#### 3.7.1. Saturation Transfer Difference (STD)

Saturation transfer difference NMR is a method for studying host-ligand interactions. Ligand peak intensities are attenuated on selective saturation of host resonances in proportion to how closely these ligands interact with the host.

Pulse sequence stddiffesgp.3 (excitation sculpted gradient pulsed) was used with a spin lock. In formulations with surfactants identified by DOSY as in micellar states, a spin lock of 30 ms was used, and when larger polymersomic structures (>100 kDa) were observed, a spin lock of 10–20 ms was used.

Selective soft pulses (1.16 mW) were set to a width of 20 Hz (50 ms) at on-resonance offset of 426 Hz for C*H*_3_ (CPC), off-resonance set to 20 KHz and further on resonance offsets of 2172 Hz (C*H*_2_N of CPC) with the off resonance set to 20 KHz.

Non-square wave pulses were used; specifically, Eburp2 was selected, as its excitation profile prevented inappropriate excitation of ligand peaks that were otherwise excited with shorter pulse lengths. Saturation points were swept for detection of the safest on-resonance setting that did not distort relative intensities but provided the most efficient saturation transfer. NS = 64, and D1 was set to 5 × T1 of the slowest relaxing compound identified in samples.

#### 3.7.2. Diffusion Ordered Spectroscopy (DOSY)

DOSY (diffusion ordered spectroscopy) is a technique for measurement of diffusion coefficients based on the delay-dependence of the signal attenuation in a pulse field gradient spin-echo experiment.

DOSY D20 and P30 values were calibrated through the acquisition of 1D experiments (ledbpgppr1d). Pulse sequence ledbpgppr2s was used with ns = 64, d1 = 5 s, D20 = 0.1 s and P30 = 0.75 ms, with 16 transients in the diffusion dimension (F1) for acquisition of 2D DOSY spectra. The software package Topspin Dynamics Center 4.3 (Bruker UK Ltd., Coventry, UK) was used to determine the diffusion coefficients using the diffusion fit function (Equation (3)) where IG = 0 is the signal intensity at a gradient strength of zero, G is the gradient strength, D is the diffusion coefficient, δ (“little delta”) is the gradient pulse duration and Δ (“big delta”) is the diffusion time.

(3) $$f\left(g\right)=I_{0}e^{-r^{2}g^{2}\delta^{2}(\Delta -\frac{\delta }{3})D}$$

Equation (3). Stejskal-Tanner equation.

Viscosity as per the Stokes-Einstein equation (Equation (4)) was corrected for by the internal reference standard (TSP), which, being water soluble, was unlikely to interact with any micelles formed.

(4) $$d_{H}=\frac{kT}{3\pi \eta D}$$

Equation (4). Stokes-Einstein equation. D_H,_ hydrodynamic diameter; k, Boltzmann distribution; T, temperature (K); η, solvent viscosity; D, diffusion coefficient.

#### 3.7.3. qNMR

Quantitation was achieved using the primary ratio method of integrals with an internal standard (TSP) at a consistent concentration (100 μM) [34]. Triplicate and interleaved experiments for each sample were acquired to evaluate reproducibility and repeatability through minimisation of noise-dependent variances.

Quantitative data were acquired with the pulse program noesygppr1d, with a scan number of 1024. D1 = 4–10 s after initial T1 relaxation experiments.

#### 3.7.4. Hydroxyethyl Cellulose (HEC) Experiments Procedure and qNMR

For the experimental formulations, standardised diffusion values of hydroxyethyl cellulose (HEC) (Merck Life Sciences, Gillingham, UK) and [CPC]_f_ had to be determined in several solvent systems to mimic those used in commercial formulations: water, 10% glycerol (G), 10% propylene glycol (PG) and a 10%/10% G and PG mix. An amount of 10 mg/mL of HEC was added to the mouthwash samples (typically 90 mg in a 9 mL mouthwash sample, prior to addition of D_2_O and TSP as per the general preparation procedure in Section 3.4; however, additional peaks assigned as HEC were selected for fitting in Topspin Dynamics Center (Bruker, Coventry, UK) as well as two components identified in the peak region due to overlap (Section 3.7.2). A range of HEC treatment concentrations (1–30 mg/mL) were initially run to measure point of gelation where changes in viscosity and relaxation properties would override any observable interaction effects. This allowed for comparison of CPC diffusion before and after introduction of HEC to deduce binding.

### 3.8. Principal Component Analysis

Principal component analysis (PCA) was undertaken on the 114 experimental formulations (Section 3.3) against the variables shown in Table 1.

## 4. Conclusions

This considerable body of work has led to a greater fundamental understanding of which excipients impact cetylpyridinium chloride (CPC) solutions and their efficacy using validated assays and the creation of a new NMR-based assessment for evaluation. Block copolymers can form mixed micelles with CPC, impacting their availability as both an antimicrobial agent and potentially affecting the results of the DRA test for full formulations. The nature of these interactions has been proposed using several NMR techniques, and there is compelling evidence that using DOSY NMR to probe CPC mobility can be used to indicate likely antimicrobial activity, even in complex formulations. The other NMR signal properties, e.g., FWHM and signal attenuation, are not as well correlated with DRA or antimicrobial activity. Experimental formulations have evidenced the impact of different excipients on the robustness of the DRA and NMR methods, and it could be proposed that where an underlying constant formulation chassis is used, there is good correlation between DRA, antimicrobial activity and DOSY NMR methods.

Whilst a diverse range of CPC-containing formulation types do not necessarily show good linear regression, the number of experimental samples and component variables used in this work still allow for multivariate analyses that show the correlation between antimicrobial activity, CPC diffusion and [CPC_f_] for all formulations and support the robustness of this NMR-based approach.

Understanding the way CPC formulations must be developed in the future to ensure high bioavailability of CPC is important to ensure efficient innovation and cost-effective formulation design. The general conclusions reached suggest that avoiding all ionic interactions and using non-ionic excipients in formulations are beneficial for [CPC]_f_, and care must be taken when adding emulsifiers, as they can have a pronounced effect on CPC mixed micelle size and hence availability. Alongside the development of the HEC-based DRA mimic model and absolute CPC diffusion, which enables rapid and cost-effective prediction of antimicrobial activity, the use of a variety of complimentary NMR methods has further allowed for the determination of a library of deleterious and beneficial excipients when designing any new CPC-based antibacterial formulation.

## Figures and Tables

**Table 1:** Labels assigned to variables in PCA matrix.

Label	Component	Label	Component
**A**	Tego betaine	**R**	Sodium Benzoate
**B**	Cremaphor RH 60	**S**	Benzoic Acid
**C**	Pluronic F108	**T**	IPMP
**D**	Polaxomer 188 Micronised	**U**	Xylitol
**E**	Polaxomer 407	**V**	Stevia
**F**	Xanthan Gum	**W**	Sucralose
**G**	Methyl parahydroxybenzoate	**Y**	Cratos EU FlavourOptamint Pallas Symrise
**H**	Propyl hydroxybenzoate, Propyl paraben	**Y**	PVPPlasdone
**I**	Sodium salt of Methyl paraben	**Z**	Guar Gum
**J**	Sodium Salt of Propyl paraben	**AA**	Potassium sorbate
**K**	Potassium sorbate	**AB**	Chitosan
**CPC Conc.**	Cetylpyridinium Chloride	**Diff**	Diffusion Coefficient (Log_10_)
**M**	Zinc Chloride/Lactate/Gluconate	**MK**	Micro Kill
**N**	Zinc citrate	**Diff Change**	Diff. Coeff. Difference ± HEC
**O**	Citric acid	**DRA**	DRA
**P**	Sodium Fluoride	**CPC Free**	CPC Mass mg/mL
**Q**	Flavour 508016T Mint Fizz protection		

Data were processed using Unscrambler X version 10.3 (Camo Software AS, Oslo, Norway). Data were first normalised according to area before PCA was undertaken with NIPALS algorithm and cross validation selected. Weighting was kept constant.

## Data Availability

The original contributions presented in the study are included in the article/Supplementary Materials, further inquiries can be directed to the corresponding author/s.

## Supplementary Materials

The following supporting information can be downloaded at: https://www.mdpi.com/article/10.3390/ph17121570/s1. Figure S1: Structure of CPC and block copolymers with NMR structural assignment; Figure S2: Flavourings and preservatives used in formulations; Figure S3: Addition of 0.025 M PBS in the presence of 0.07& *w*/*w* CPC; Figure S4: PCA Scores plot for all experimental formulations. The groupings reflect the diverse range of experimental chassis provided by Haleon PLC. Table S1: Raw data.
