# Supplementary material for: Modelling of Cetylpyridinium Chloride Availability in Complex Mixtures for the Prediction of Anti-Microbial Activity Using Diffusion Ordered Spectroscopy, Saturation Transfer Difference and 1D NMR
Source: Pharmaceuticals (Basel). 2024 Nov 22;17(12):1570. doi: 10.3390/ph17121570 (PMC11679348; doi:10.3390/ph17121570)
Supplement: Supplementary file 1 [file pharmaceuticals-17-01570-s001.zip › pharmaceuticals-3250989-supplementary.pdf]

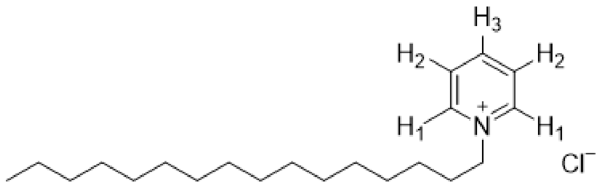

Cetylpyridinium chloride (CPC)

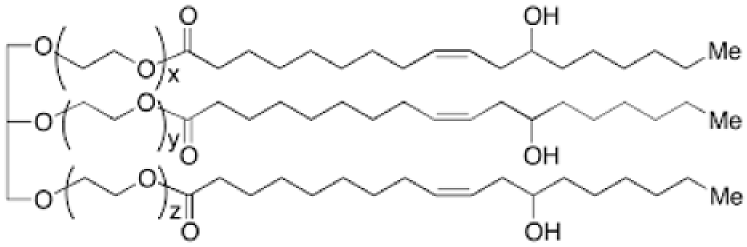

Cremophor (4500Da)

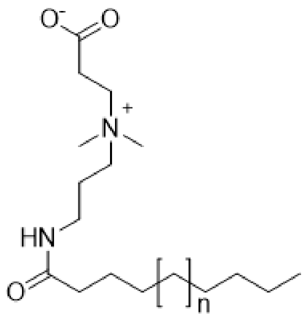

Tegobetaine (P407) (500Da)

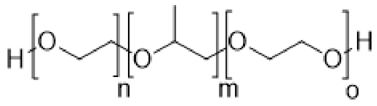

Figure S1. Structure of CPC (with assigned protons used throughout paper) and the block co-polymers.

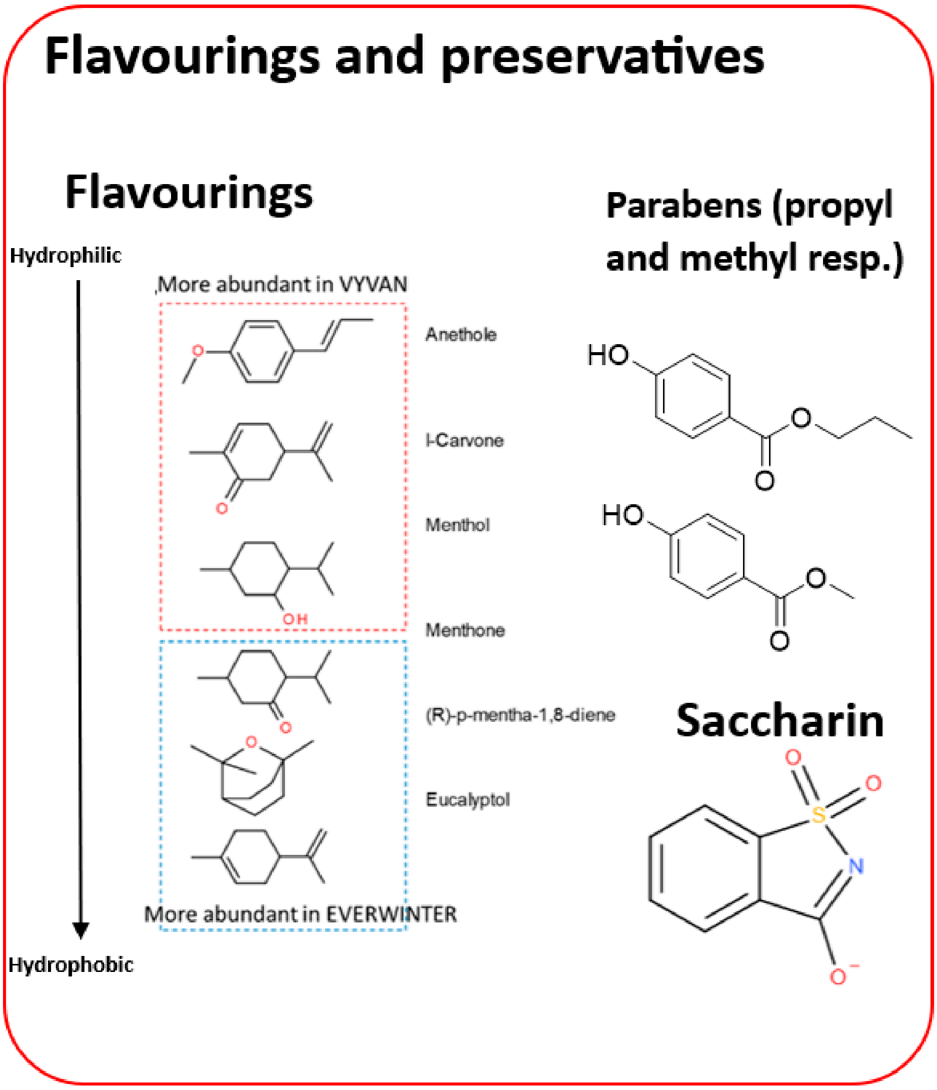

Figure S2. Flavourings and preservatives used in formulations.

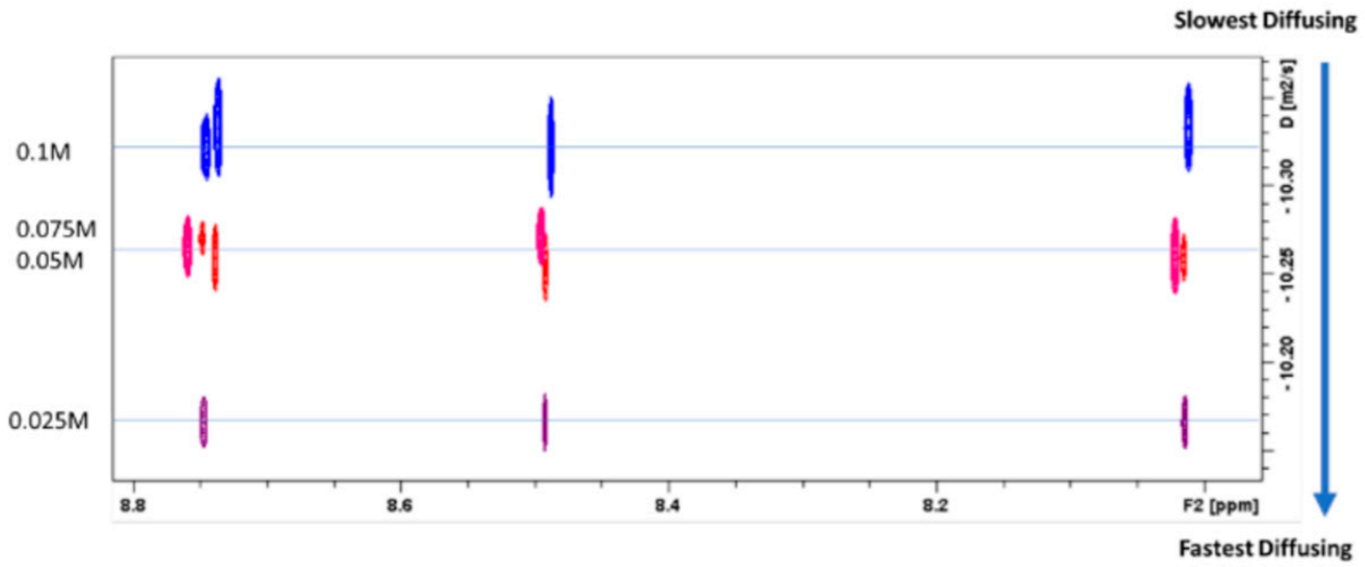

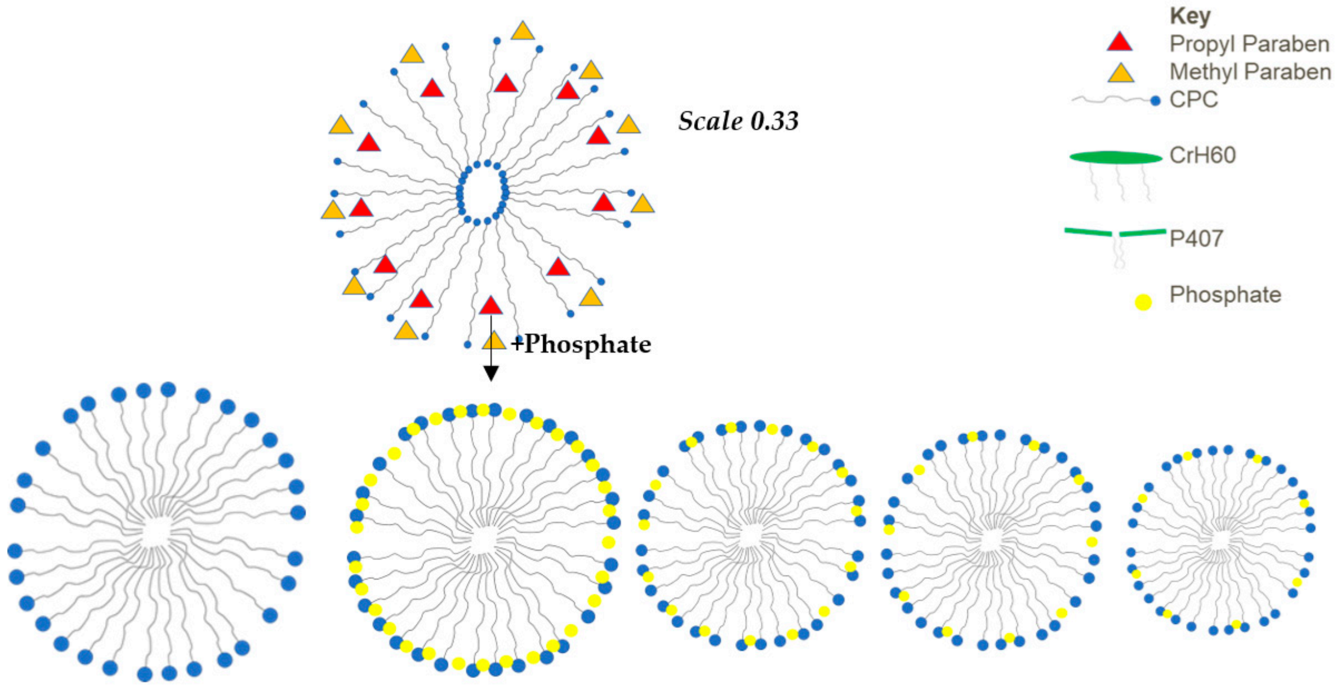

Figure S3. Addition of 0.025M PBS in the presence of 0.07& w/w CPC.

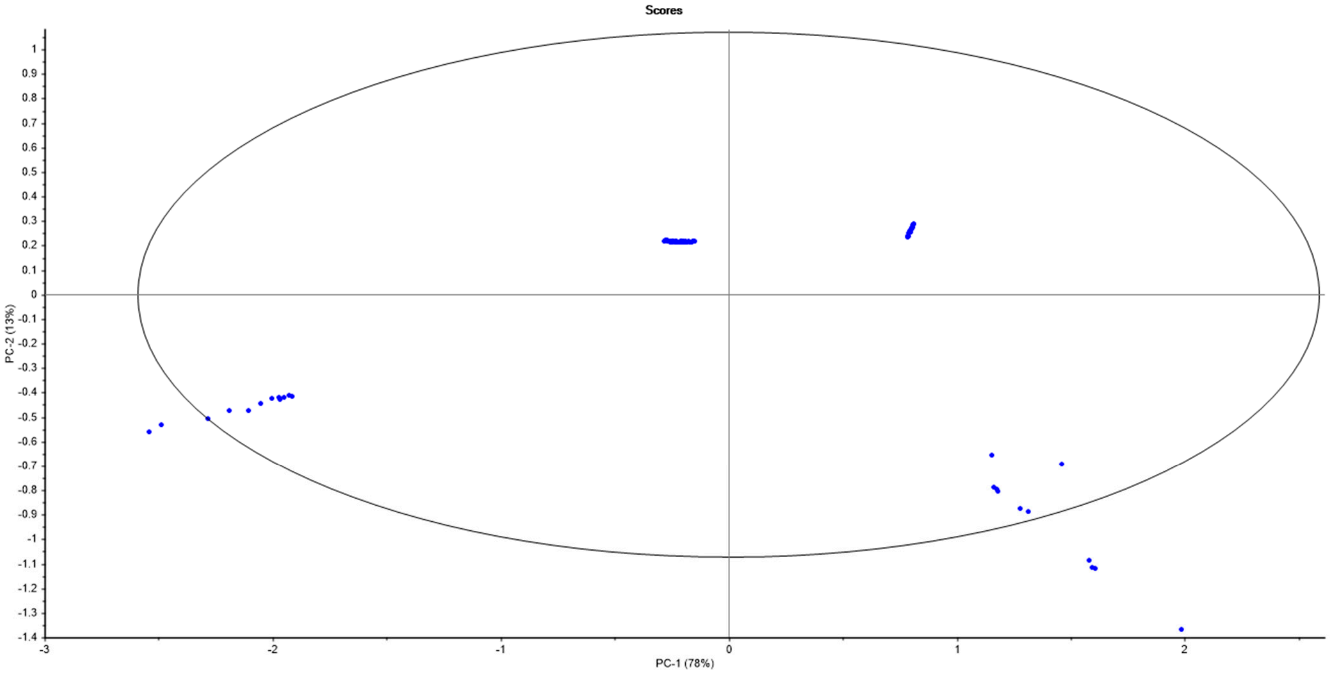

Figure S4. PCA Scores plot for all experimental formulations. The groupings reflect the diverse range of experimental chassis provided by Haleon PLC.
